# Identification of the microRNA alterations in extracellular vesicles derived from human haemorrhoids

**DOI:** 10.1113/EP090549

**Published:** 2023-01-09

**Authors:** Kaijing Wang, Yuanyuan Zhang, Xiaoxue Ma, Xinyu Ge, Yewei Deng

**Affiliations:** ^1^ Department of Hepatological Surgery General Surgery Shanghai East Hospital Tongji University School of Medicine Shanghai China; ^2^ Department of Colorectal Surgery General Surgery Shanghai East Hospital Tongji University School of Medicine Shanghai China; ^3^ Translational Medical Center for Stem Cell Therapy and Institute for Regenerative Medicine Shanghai East Hospital Tongji University School of Medicine Shanghai China; ^4^ Department of Thoracic Surgery Shanghai East Hospital Tongji University School of Medicine Shanghai China

**Keywords:** extracellular vesicles, haemorrhoids, microRNA profile

## Abstract

Extracellular vesicles (EVs) play important roles in many pathophysiologies as cell‐to‐cell communication vehicles. However, the features and potential functions of the EVs in haemorrhoids remain unclear. Therefore, we performed microRNA (miRNA) microarray analysis in EVs derived from haemorrhoid tissue to identify the profile of miRNAs in these EVs and predict their potential functions. We obtained typical EVs from both haemorrhoid and control tissues. Microarray analysis identified 447 miRNAs with significant differential expresssion (DE): 245 upregulated and 202 downregulated. The top three upregulated miRNAs in haemorrhoid EVs (Hae‐EVs), namely miR‐6741‐3p, miR‐6834‐3p and miR‐4254, were detected by RT‐qPCR in both Hae‐EVs and haemorrhoid tissues. Interestingly, we found a different expression pattern in the haemorrhoid tissues from that in Hae‐EVs. The potential target genes of these DE‐miRNAs were predicted by the miRWalk and miRDB databases. Gene ontology (GO) analysis of the target genes showed that the DE‐miRNAs contributed mainly to protein kinase activity, transcriptional activity and ubiquitin‐protein function. KEGG search found that the DE‐miRNAs might regulate the MAPK and Ras signalling pathways. These findings revealed, for the first time, the miRNA profiles in Hae‐EVs and provided potential targets and pathways involved in the pathological process.

## Abstract (highlights)

- **What is the central question of this study?**
  What are the morphological features and microRNA (miRNA) expression features of extracellular vesicles (EVs) from haemorrhoids (Hae‐EVs) and normal tissues? What are the potential functions of the differentially expressed (DE) miRNAs in Hae‐EVs?
- **What is the main finding and its importance?**
  We present, for the first time, the morphological features and miRNA profile of human Hae‐EVs. Four hundred and forty‐seven significant DE‐miRNAs were identified. Gene ontology and pathway analysis of the DE‐miRNAs indicated diverse roles of the Hae‐EVs through different pathways. Our findings provide EV‐based pathological features and the underlying mechanism of haemorrhoids.

## 1 INTRODUCTION

Haemorrhoids are a common and recurrent anorectal condition and represent a considerable medical and socioeconomic burden (Lohsiriwat et al., 2012). Common symptoms of haemorrhoids include bleeding, pain, pruritus, faecal seepage, prolapse and discharge of mucus (Ganz, 2013), which greatly impair the quality of life of patients. The prevalence of haemorrhoids increases with age and has been reported to be as high as 30−39% in the adult general population (Acheson & Scholefield, 2008; Loder et al., 1994), and can be ≤86% prevalence in some reports (Haas et al., 1983). However, the mechanism of haemorrhoids is still unclear.

Haemorrhoids are abnormal anal vascular cushions filled with blood at the junction of the rectum and the anus. Based on their location, they can be classified as internal, external or mixed haemorrhoids (Lohsiriwat, 2013; Mounsey et al., 2011). The causes of haemorrhoids are extremely complex. A number of risk factors have been suggested, including the human erect posture, obesity, low dietary fibre intake, sedentary lifestyle, spending excess time on the toilet, strenuous lifting, constipation, diarrhoea and pelvic floor dysfunction, with several being reported controversially. A well‐powered genome‐wide association study (GWAS) meta‐analysis found a genetic component that predisposes to smooth muscle, epithelial and connective tissue dysfunction in haemorrhoids (Zheng et al., 2021). Abnormal venodilatation and destructive changes in the supporting connective tissue within the anal cushion are characteristic features of haemorrhoids (Loder et al., 1994; Lohsiriwat et al., 2012). Matrix metalloproteinases, which regulate extracellular proteins and tissue remodelling, have been found to be elevated in patients with haemorrhoids (Serra et al., 2016). Abnormalities in the quality and quantity of collagen in haemorrhoid patients could result in decreased mechanical stability (Nasseri et al., 2015; Willis et al., 2010). Constipation leads to chronic straining and hard stools, resulting in degeneration of the supportive tissue in the anal canal and distal displacement of the anal cushion (Talley et al., 2009). Despite being prevalent, the pathophysiology of this condition remains elusive, with few reports on the mechanisms of haemorrhoids.

Extracellular vesicles (EVs) are nanosized cell‐derived membranous vesicles that exist throughout the body (Nieuwland et al., 2018). Previous studies have demonstrated the vital roles of EVs in numerous biological functions, including cancer progression and metastasis (Tkach & Thery, 2016), wound healing (Cabral et al., 2018), angiogenesis (Todorova et al., 2017) and immunoregulation (Maas et al., 2017). Investigations of the EVs in haemorrhoids (Hae‐EVs) might provide new clues to help elucidate the pathological features and development. As vital cell‐to‐cell communication vehicles (Colombo et al., 2014), EVs can deliver various substances, such as proteins, lipids and RNAs, to recipient cells and regulate their functions (Abels & Breakefield, 2016; Crewe et al., 2018). A key role of microRNA (miRNA) cargos from tissue EVs has been demonstrated in the process of cardiac disease (Ge et al., 2021). Investigation of the EVs and their contents in haemorrhoids might help to disclose the pathological features and provide new treatment strategies.

As far as we are aware, there is little information regarding the features and role of EVs in haemorrhoids. Therefore, we performed miRNA microarray analysis in EVs derived from haemorrhoid tissue (Hae‐tis) to identify the profile of miRNAs in these EVs, and we evaluated the function of Hae‐EVs in development of the pathology.

## 2 METHODS

### 2.1 Ethical approval

All specimens were collected from patients referred to Shanghai East Hospital for surgical management. The control rectal mucosa and submucosa were obtained from eight low rectal cancer patients receiving anterior resection or abdominoperineal resection without previous pelvic radiotherapy. The haemorrhoid samples were rectal mucosa and submucosa tissues adjacent to haemorrhoids from eight haemorrhoid patients undergoing procedures for prolapsed haemorrhoids. Samples were stored at −80°C until use. This study conformed to the standards set by the latest revision of the *Declaration of Helsinki*, except for registration in a database, and was approved by the institutional review board of Shanghai East Hospital (2019tjdx39). Written informed consent was obtained from the participants.

### 2.2 Extracellular vesicle isolation

Extracellular vesicle isolation was performed according to a previous study (Ge et al., 2019). Briefly, the tissues were washed with PBS, then cut into small pieces. The tissue mass was incubated in 4 ml of type IV collagenase (1.5 mg/ml; Worthington Biochemical, USA) and DNase (60 U/ml; Sigma, USA) at 37°C for 30 min. The digested tissues were centrifuged at 300*g* for 5 min at 4°C to remove the tissues and cells. The supernatant was centrifuged at 2,000*g* for 10 min, then at 10,000*g* for 10 min at 4°C. The supernatant was centrifuged at 120,000 g for 120 min at 4°C (Optima L‐100XP Ultracentrifuge, Beckman Coulter). The EVs were obtained after washing with PBS.

### 2.3 Extracellular vesicle quantification

The particle size of the EVs was detected using the Nanoparticle Tracking Analysis (NTA) technique supported by VivalCell, Shanghai with three replicates.

### 2.4 Transmission electron microscopy

The freshly isolated EVs were fixed with 2.5% glutaraldehyde and loaded onto 200 Mesh carbon‐coated formvar grids for 5 min. Then 2% phosphotungstic acid was used for EV staining (5 min at room temperature). Extracellular vesicles were detected under a transmission electron microscope (Hitachi, HT7700), and we found a typical saucer‐shaped appearance.

### 2.5 Microarray and data analysis

The human miRNA microarray experiment and data analysis of the 16 samples were performed by OE Biotechnology (Shanghai, China). Total RNA was isolated using TRIzol reagent (Invitrogen, MA, USA). The concentration and purity of RNAs were determined with a NanoDrop ND‐2000 spectrophotometer (Thermo, Waltham, MA, USA), and the integrity of the RNA was assessed using an Agilent Bioanalyzer 2100 (Agilent Technologies). The sample labelling, microarray hybridization and washing were performed based on the manufacturer's standard protocols. Briefly, total RNA was dephosphorylated, denatured, then labelled with cyanine‐3‐CTP. The labelled RNAs were hybridized onto the microarray after purification. After washing, the arrays were scanned with an Agilent Scanner G2505C (Agilent Technologies).

The raw data were acquired using Feature Extraction software (v.10.7.1.1; Agilent Technologies). Genespring software (v.14.8, Agilent Technologies) was used for the basic analysis. Initially, we normalized the raw data based on the quantile algorithm. The probes with all the samples in one group flagged ‘detected’ were selected for further analysis. Differentially expressed (DE) miRNAs were identified according to the fold change and *P*‐value (fold change ≥ 2.0 and *P*‐value ≤ 0.05) calculated by Student's unpaired *t*‐test. The target genes of the DE‐miRNAs were predicted by two databases (miRWalk and miRDB). In the miRDB database, the predicted genes with scores >80 were selected. Gene ontology (GO) and KEGG pathway enrichment analyses were applied to determine the roles of these target genes. Hierarchical clustering was performed to show the expression pattern of distinguishable miRNAs among samples.

### 2.6 Western blot

The EV samples were lysed and extracted with RIPA buffer. Proteins were loaded on 10% SDS‐PAGE gels, then transferred to polyvinylidene difluoride membranes. After blocking for 1 h at room temperature, the membranes were incubated overnight at 4°C with primary antibodies directed to CD63 (Proteintech, 25682‐1‐AP, 1:1,000 dilution), CD9 (Proteintech, 20597‐1‐AP, 1:1,000 dilution) and TSG101 (Proteintech, 28283‐1‐AP, 1:1,000 dilution). After three washes with TBST, the membranes were incubated with the corresponding secondary antibodies (Beyotime, A0208 or A0216, 1:1,000 dilution) for 1 h, and the bands were detected by chemiluminescence (Pierce, USA).

### 2.7 Quantitative real‐time polymerase chain reaction analysis

Total RNA was isolated using TRIzol reagent (Invitrogen, USA). Reverse transcription was performed using the microRNA reverse transcription kit (Takara Bio, Otsu, Japan). RT‐qPCR was performed in a 10 μl reaction system containing forward/reverse primers, complementary DNA and SYBR Green MasterMix (Takara Bio) with three replicates. The miRNA levels were normalized to the endogenous control U6 small nuclear RNA. The relative expression of these RNAs was calculated using the 2^−∆∆^ *^CT^* method. The primers used in the study are listed in Table 1.

**TABLE 1:** The primers used in the present study.

**MicroRNA**	**Forward primer**	**Reverse primer**
miR‐6741‐3p	TCGGCTCTCTCCCTCACCCTAG	Universal
miR‐6834‐3p	TATGTCCCATCCCTCCATCA	Universal
miR‐4254	GCCTGGAGCTACTCCACCATCTC	Universal
U6	CTCGCTTCGGCAGCACA	Universal

### 2.8 Statistical analysis

The data were expressed as the mean ± SD. Anderson–Darling test was used for assessment of the normality of the data. Student's unpaired *t*‐test was used for the comparison between the two groups. GraphPad Prism 8.0 (GraphPad Software, San Diego, CA, USA) was used for data analysis. A *P*‐value of <0.05 was considered statistically significant.

## 3 RESULTS

### 3.1 Characteristics of the Hae‐EVs

**FIGURE 1:**
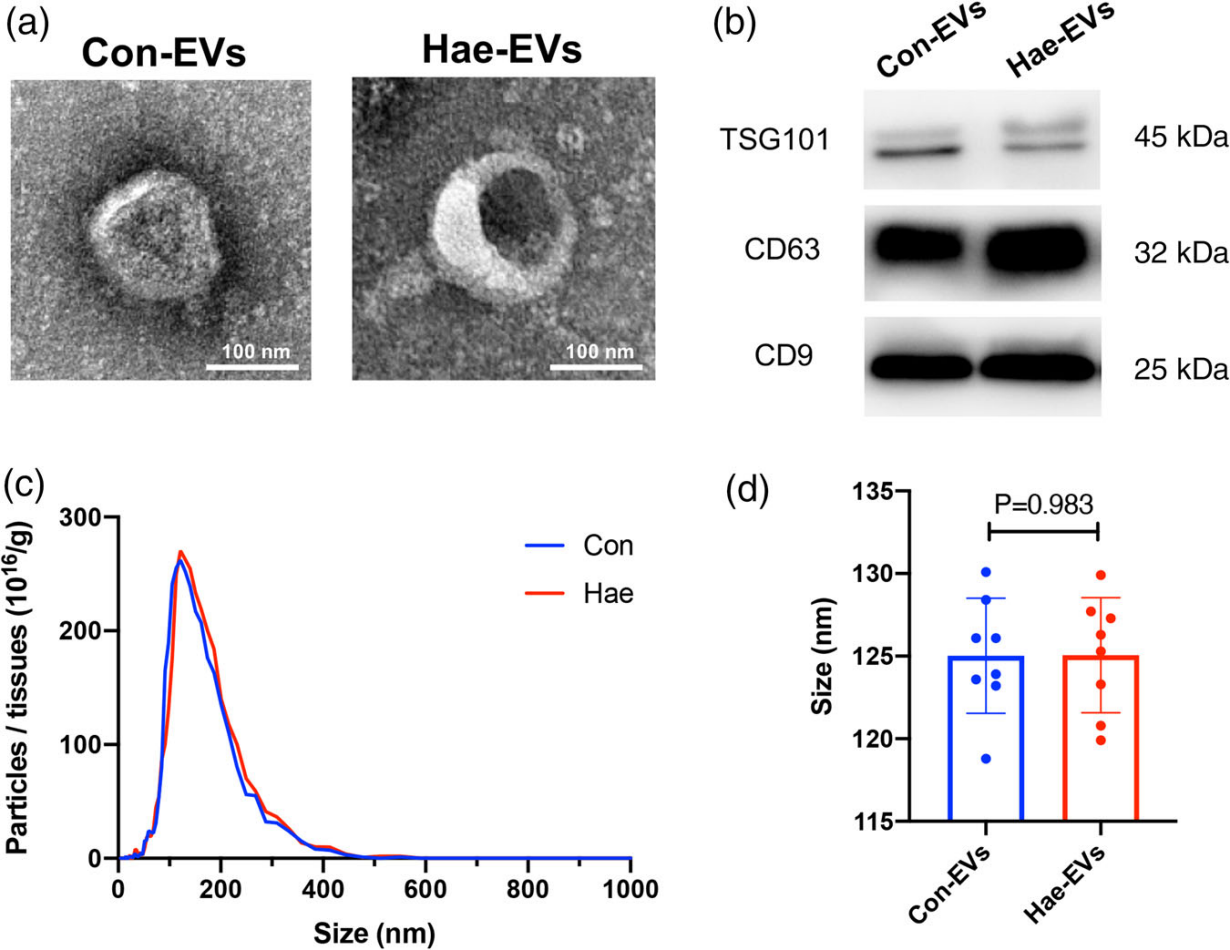
Characterization of cardiac extracellular vesicles (EVs). (a) A representative transmission electron microscope image of control (Con‐EVs) and haemorrhoid EVs (Hae‐EVs). Scale bars: 100 nm. (b) Western blot showing the typical exosomal markers (Tsg101, CD63 and CD9) in the Con‐EVs and Hae‐EVs. (c) The particle size distribution of the EVs was measured using nanoparticle tracking analysis. (d) The size of EVs detected by nanoparticle tracking analysis in (c); *n* = 8.

The typical morphology of EVs from control and haemorrhoid mucosa was captured under the transmission electron microscope (Figure 1a). As shown in Figure 1b, typical exosome markers, namely TSG101, CD63 and CD9, were expressed in the control (Con‐EVs) and haemorrhoid EVs (Hae‐EVs). The size distribution of the EVs is shown in Figure 1c, and there was no difference between the size of the Con‐EVs and Hae‐EVs (Figure 1d).

### 3.2 Differential miRNA profiles in the Con‐EVs and Hae‐EVs

**FIGURE 2:**
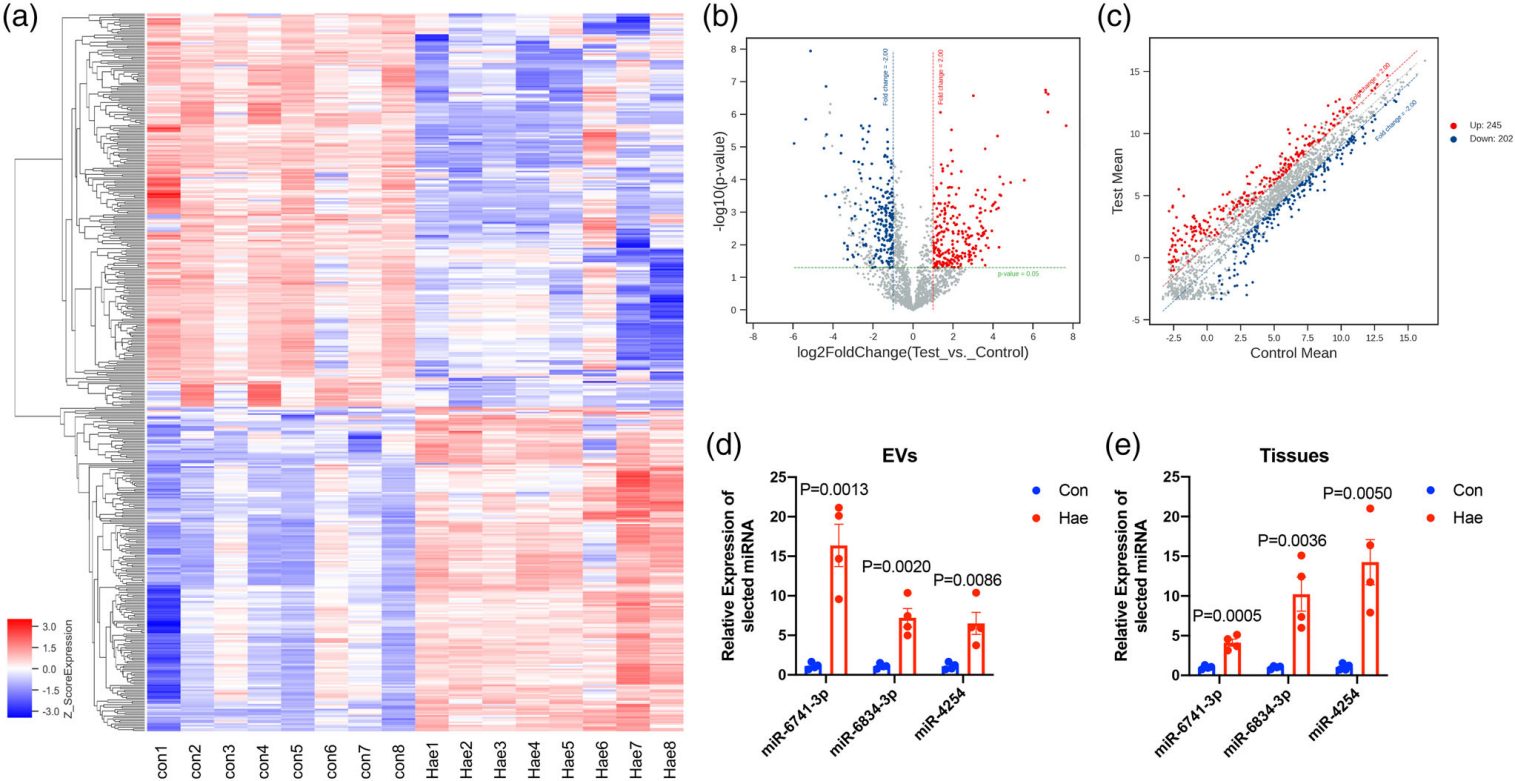
Differential expression profile of microRNAs (miRNAs) in the control (Con‐EVs) and haemorrhoid extracellular vesicles (Hae‐EVs). (a) Clustered heat map of the differentially expressed microRNAs (DE‐miRNAs). (b) Volcano plot of the DE‐miRNAs. Significantly up‐ and downregulated miRNAs are shown as red and green dots, respectively. (c) Scatter plot of the DE‐miRNAs. Significantly up‐ and downregulated miRNAs are shown as red and green dots, respectively. (d) The top three upregulated miRNAs in Hae‐EVs, namely miR‐6741‐3p, miR‐6834‐3p and miR‐4254, were detected by RT‐qPCR to test the microarray data; *n* = 4. (e) RT‐qPCR was performed to detect the top three upregulated miRNAs in Hae‐EVs, namely miR‐6741‐3p, miR‐6834‐3p and miR‐4254, in human tissues (*n* = 4).

Next, by using a miRNA microarray test, a total of 447 significantly DE‐miRNAs, comprising 245 upregulated and 202 downregulated miRNAs, were identified in the Hae‐EVs compared with the Con‐EVs. A heat map of the DE‐miRNAs is presented in Figure 2a. Significantly up‐ and downregulated miRNAs are shown as red and green dots on a volcano plot (Figure 2b) and a scatter plot (Figure 2c). The top 10 upregulated and downregulated miRNAs are listed in Tables 2 and 3, respectively. To confirm the differential expression of miRNAs in our microarray data, the top three upregulated miRNAs in Hae‐EVs, namely miR‐6741‐3p, miR‐6834‐3p and miR‐4254, were detected by RT‐qPCR, which was consistent with the microarray results (Figure 2d). Furthermore, we tested the expression of the selected miRNAs in the haemorrhoid and control tissues (Figure 2e). Interestingly, among the top three upregulated miRNAs in Hae‐EVs, miR‐6741‐3p showed the highest expression in Hae‐EVs but the lowest expression in the tissues. This finding suggested that miR‐6741‐3p was more obviously enriched in the Hae‐EVs.

**TABLE 2:** Top 10 upregulated microRNAs in extracellular vesicles from haemorrhoid patients versus control subjects.

**Probe ID**	***P*‐value**	**Fold change**	**Regulation**
hsa‐miR‐6741‐3p	2.23 × 10^−6^	201.772234	Up
hsa‐miR‐6834‐3p	2.40 × 10^−7^	107.821226	Up
hsa‐miR‐4254	8.52 × 10^−7^	107.69946	Up
hsa‐miR‐6804‐3p	2.12 × 10^−7^	99.9014845	Up
hsa‐miR‐744‐3p	1.78 × 10^−7^	99.2044693	Up
hsa‐miR‐8485	0.00010473	47.233107	Up
hsa‐miR‐299‐5p	0.00012194	29.4809102	Up
hsa‐miR‐4636	0.0001369	22.828314	Up
hsa‐miR‐3175	0.00030779	21.0114743	Up
hsa‐miR‐4658	8.24 × 10^−4^	20.3270224	Up

**TABLE 3:** Top 10 downregulated miRNAs in extracellular vesicles from haemorrhoid patients versus control subjects.

**Probe ID**	***P*‐value**	**FoldChange**	**Regulation**
hsa‐miR‐548t‐5p	7.74 × 10^−6^	−62.203728	Down
hsa‐miR‐323b‐5p	1.42 × 10^−6^	−41.541603	Down
hsa‐miR‐1322	1.14 × 10^−8^	−35.106549	Down
hsa‐miR‐3928‐5p	1.11 × 10^−5^	−22.050293	Down
hsa‐miR‐346	1.40 × 10^−7^	−20.574954	Down
hsa‐miR‐4704‐5p	0.00032414	−20.426978	Down
hsa‐miR‐1913	4.11 × 10^−6^	−20.145463	Down
hsa‐miR‐876‐3p	0.00028955	−15.012559	Down
hsa‐miR‐4460	0.0006992	−12.891665	Down
hsa‐miR‐892a	1.55 × 10^−5^	−12.178714	Down

### 3.3 Differential miRNA expression between tissue and EVs in the haemorrhoid patients

**FIGURE 3:**
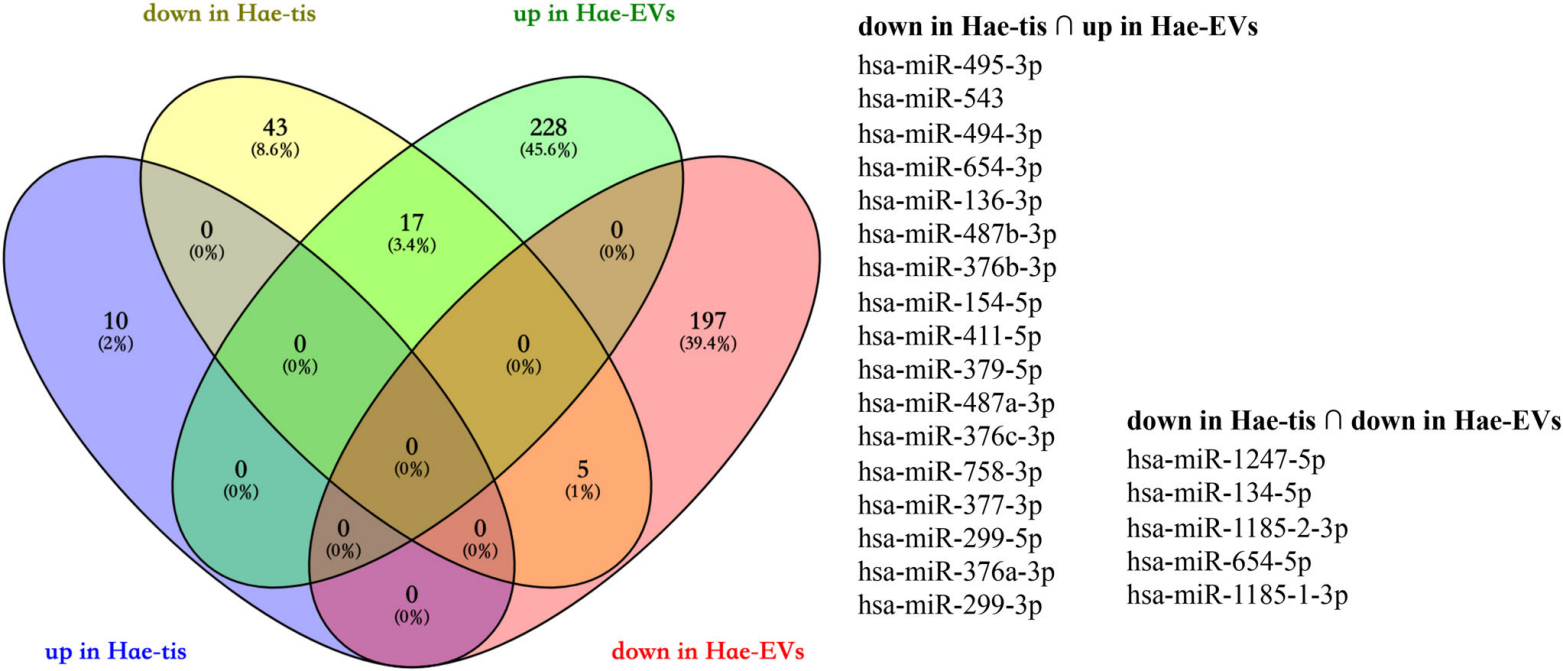
Differential expression of microRNAs (miRNAs) in the haemorrhoid tissues (Hae‐tissues) and haemorrhoid extracellular vesicles (Hae‐EVs). Venn diagram showing 17 miRNAs that were significantly upregulated in Hae‐tis and downregulated in Hae‐EVs and five miRNAs that were significantly downregulated in Hae‐tis and also downregulated in Hae‐EVs.

From the expression results in previous studies (Song et al., 2020; Wang et al., 2019), we obtained 10 upregulated miRNAs and 65 downregulated miRNAs in Hae‐tiss compared with those in control samples (Figure 3). After Venn analysis of these results and our data, we found 17 miRNAs that were significantly upregulated in Hae‐tis and downregulated in Hae‐EVs, such as hsa‐miR‐495‐3p, hsa‐miR‐543, hsa‐miR‐494‐3p, hsa‐miR‐654‐3p and hsa‐miR‐136‐3p, in addition to five miRNAs that were significantly downregulated in Hae‐tis and downregulated in Hae‐EVs, namely hsa‐miR‐1247‐5p, hsa‐miR‐134‐5p, hsa‐miR‐1185‐2‐3p, hsa‐miR‐654‐5p and hsa‐miR‐1185‐1‐3p.

### 3.4 Target prediction of the DE‐miRNAs

**FIGURE 4:**
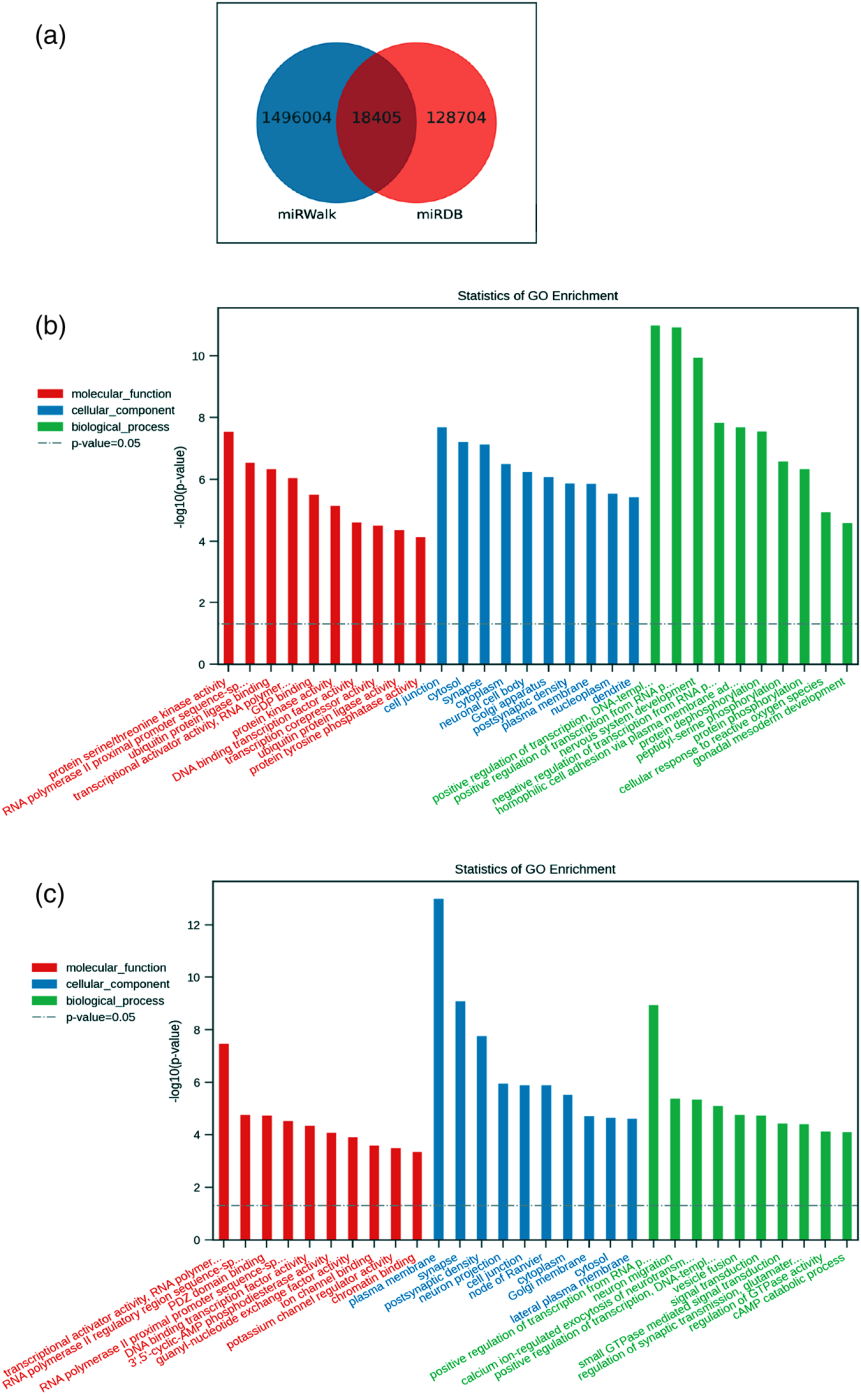
Predicted functions of the differentially expressed microRNAs (DE‐miRNAs) in the extracellular vesicles (EVs). Gene ontology (GO) analysis including (a) biological processes, (b) cell components and (c) molecular functions of the downregulated miRNAs. Gene ontology analysis including (d) biological processes, (e) cell components and (f) molecular functions of the upregulated miRNAs.

We found 1,496,004 target genes in the miRWalk database and 128,704 genes in the miRDB database. From the intersection, we obtained 18,405 target genes (Figure 4a). The top five upregulated and downregulated miRNAs with the highest miRDB score are presented in Table 4.

**TABLE 4:** Target genes of the top five up‐ and downregulated microRNAs.

Gene ID	Target gene	miRWalk_BindingSites	miRDB_Score
Top five upregulated			
hsa‐miR‐6741‐3p	*UBQLN1*	3425–3477	83.2318
hsa‐miR‐6834‐3p	*ZFAND1*	1137–1175; 1172–1196	94.3588632
hsa‐miR‐4254	*GPD1*	1478–1504; 1547–1573	93.0146692
hsa‐miR‐6804‐3p	*PPP4R1*	3043–3109	94.307589
hsa‐miR‐744‐3p	*NDUFA5*	1697–1715; 1769–1787; 1818–1849	84.8287893
Top five downregulated			
hsa‐miR‐548t‐5p	*ATG16L1*	2285–2310	93.77388
hsa‐miR‐323b‐5p	*SERF2*	1959–1983; 2070–2094	93.40676
hsa‐miR‐1322	*MIER1*	2894–2908; 3038–3052; 3115–3129	90.03152
hsa‐miR‐3928‐5p	*CACNA1E*	8355–8410|13731–13753; 8412–8467|13788–13810; 8541–8596	97.0246725
hsa‐miR‐346	*NXPH1*	2392–2431	96.2153598

### 3.5 Gene ontology analysis of the DE‐miRNA targets

The potential functions of the upregulated miRNAs (Figure 4b) and downregulated miRNAs (Figure 4c) were assessed based on GO analysis of the target genes. The GO analysis of molecular function showed that the upregulated miRNAs contributed mostly to protein kinase activity, transcriptional activity and ubiquitin‐protein function. The GO cell component terms of the upregulated miRNAs were significantly enriched at the site of cell junctions. The GO analysis of biological processes indicated that the upregulated miRNAs were primarily involved in the regulation of transcription.

From the GO enrichment results of DE‐miRNAs in Figure 4c, we found that the downregulated miRNAs were associated mainly with transcriptional activator activity. The major GO cell component terms of the downregulated miRNAs were related to the plasma membrane. The GO analysis of biological processes indicated that the downregulated miRNAs were mainly related to regulation of transcription.

### 3.6 KEGG pathway analysis of the DE‐miRNA targets

**FIGURE 5:**
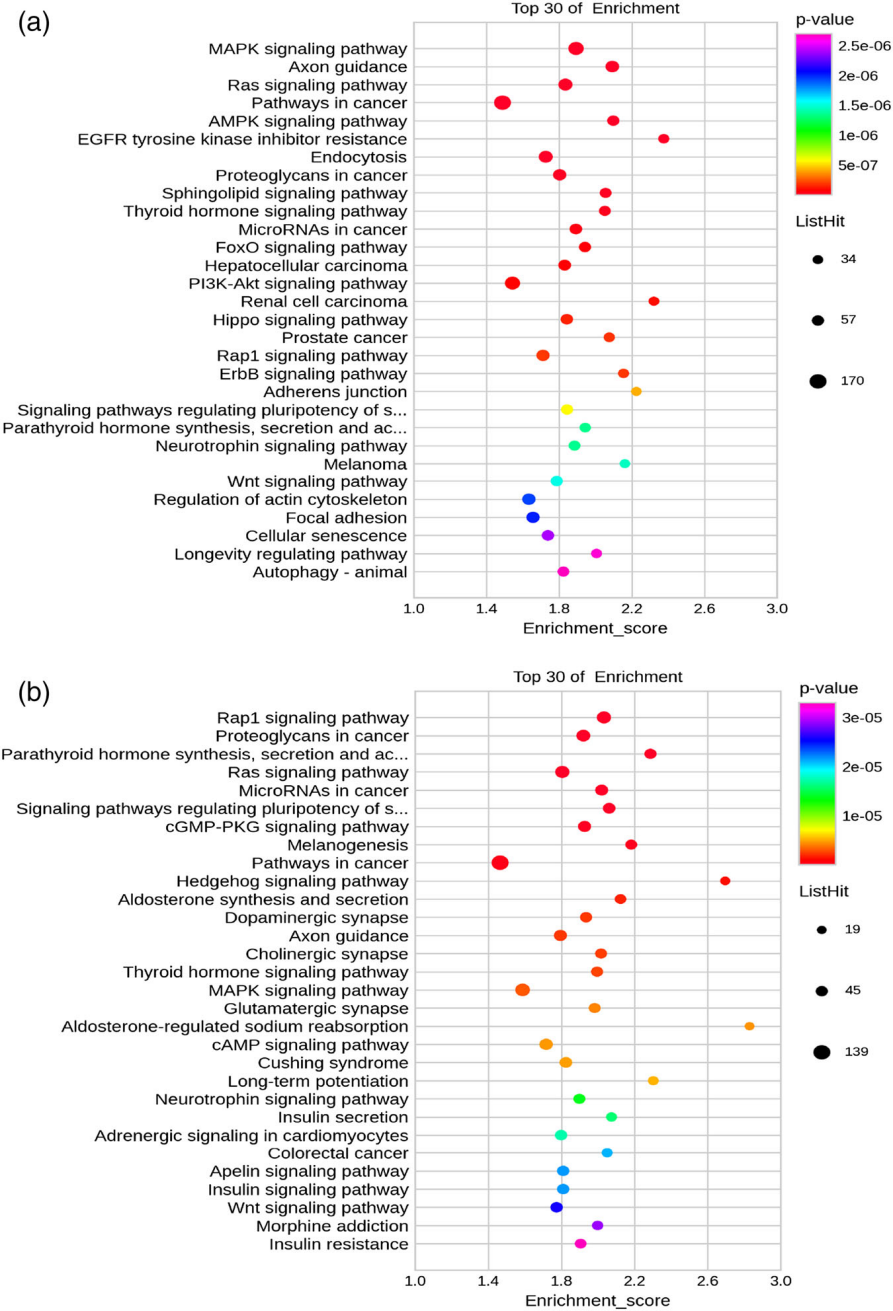
Predicted pathways of the differentially expressed microRNAs (DE‐miRNAs) in the extracellular vesicles (EVs). (a) Pathway analysis of the downregulated miRNAs. (b) Pathway analysis of the upregulated miRNAs.

We also performed KEGG pathway analysis using the targets of the DE‐miRNAs (Figure 5). The top 30 significantly upregulated pathways of miRNAs are shown in Figure 5a. The top three pathways according to the *P*‐values were the MAPK signalling pathway, axon guidance and the Ras signalling pathway. Other significant pathways, such as the AMPK, PI3K‐Akt, Hippo and Wnt signalling pathways and autophagy were also involved. Regarding the pathway analysis of downregulated miRNAs (Figure 5b), the top three significantly downregulated pathways were the Rap1 signalling pathway, proteoglycans in cancer, and the pathway of parathyroid hormone synthesis, secretion and action. Other important pathways included the MAPK, cAMP and Wnt signalling pathways.

## 4 DISCUSSION

As a very common anorectal condition, haemorrhoids beset millions of people around the world, with a lack of curative treatments. Given the relative lack of clinical and molecular research, our knowledge of haemorrhoids is very limited. Increasing evidence has confirmed the vital roles of EVs in the diagnosis, treatment and pathogenesis of various diseases. Little is known about the content and function of EVs in haemorrhoids. In the present study, we identified the specific expression profile of miRNAs in EVs derived from human Hae‐tiss and provided their potential functions and pathways. Interestingly, we also found a different expression pattern between the tissue and EVs of haemorrhoids. Our study revealed, for the first time, the alteration of miRNAs in Hae‐EVs compared with Con‐EVs and provided potential fundamental clues to their roles in the pathological development of haemorrhoids.

Extracellular vesicles derived from the circulation or tissues have been confirmed to have various functions in both clinical settings and in vivo animal models. The present study disclosed the features of EVs from human haemorrhoids. There were no obvious differences in the morphology and concentration between the EVs from control and Hae‐tiss. The exosome marker of TSG101 was more highly expressed in Con‐EVs compared with Hae‐EVs. Despite the physical resemblance, the miRNA cargos between Con‐EVs and Hae‐EVs are different, which reminds us of the potential role of EVs and their miRNA cargos in the pathogenesis of haemorrhoids. As important cargos in EVs, miRNAs have been reported to participate in the development of haemorrhoids. For example, the downregulation of miR‐4729 has been confirmed in haemorrhoid vascular endothelial cells and contributes to vascular proliferation (Liu et al., 2021). Moreover, miR‐412‐5p can regulate angiogenesis in Hae‐tis by targeting Xpo1 (Wang et al., 2019). Therefore, we performed a microarray of miRNAs to show the miRNA profile in the EVs from control and Hae‐tiss and found 447 significantly DE‐miRNAs, including 245 upregulated and 202 downregulated miRNAs in the Hae‐EVs compared with the Con‐EVs. The top three upregulated miRNAs in Hae‐EVs, namely miR‐6741‐3p, miR‐6834‐3p and miR‐4254, were also confirmed by RT‐qPCR. Their expressions were also investigated in the haemorrhoid and control tissues. Interestingly, we found a different pattern of expression in the Hae‐tiss from that in Hae‐EVs. Likewise, combined with the previously reported miRNA expression data in the haemorrhoid and control tissues, we found that the miRNA expression pattern in Hae‐EVs and tissues was not always the same. Some miRNAs might be enriched in EVs, whereas some are the opposite, such as the 17 miRNAs that were significantly upregulated in Hae‐tis and downregulated in Hae‐EVs shown in our study. Recent study has highlighted the use of EVs for early diagnosis of cancers. The present study might provide new targets and ideas for future mechanistic studies, and new thoughts will be inspired, such as using haemorrhoid‐EVs as biomarkers for evaluation of disease severity and prediction of recurrence.

To identify the potential function of Hae‐EVs, we predicted the target genes of the DE‐miRNAs. As the most highly expressed DE‐miRNA, miR‐6741‐3p was predicted to combine the 3′‐UTR of *UBQLN1*. A previous study reported that the ubiquitin ligase HERC3 attenuated nuclear factor‐κB‐dependent transcription by delivering the RelA subunit for degradation. Major pathological damages in haemorrhoids include local inflammation and swelling, and tissue EVs have been reported to contribute to the local and systemic inflammation (Ge et al., 2021). There is a possibility that Hae‐EV‐delivered miR‐6741‐3p enhances the local inflammation of haemorrhoids by targeting *UBQLN1*. However, further study is needed to disclose their relationship.

In the present study, we identified a total of 18,405 target genes. KEGG pathway analysis of the target genes revealed some important signalling pathways, which helped to elucidate the possible mechanisms. Mitogen‐activated protein kinases (MAPKs) are a class of highly conserved serine/threonine protein kinases in cells that transmit signals through a three‐level cascade. The MAPK pathway is involved in angiogenesis during myocardial infarction (Zhang et al., 2022). Priming of p38 MAPK activity enhanced arteriogenesis after femoral artery occlusion (Sahún‐Español et al., 2022). Vascular hyperplasia and oedema are particularly obvious in haemorrhoids (Fox et al., 2014; Sugerman, 2014). Thus, as the most significant pathway shown in our KEGG analysis, the MAPK signalling pathway is supposed to play a key role in the Hae‐EV‐induced pathological progress of haemorrhoids by targeting vascular hyperplasia. In addition, mounting evidence suggests that the MAPK signalling cascade participates in various biological responses, such as inflammation. The p38 MAPK inhibitors have shown promise for treatment of inflammatory diseases (Yong et al., 2009). Therefore, Hae‐EVs might regulate the inflammatory state in haemorrhoids by modulating the MAPK pathway. However, further studies are needed to explore the specific mechanism of these DE‐miRNAs in Hae‐EVs.

## 5 CONCLUSION

In summary, in our study we identified, for the first time, the miRNA profile of human Hae‐EVs. Gene ontology and pathway analysis of the DE‐miRNAs indicated diverse roles of the Hae‐EVs through different pathways. The Hae‐EVs and the miRNA cargos might regulate target gene expression, leading to an imbalance in certain signal transduction pathways, thus contributing to the development of haemorrhoids. Further functional studies are expected to confirm the relationship and underlying mechanism based on these findings.

## AUTHOR CONTRIBUTIONS

Xinyu Ge and Yewei Deng conceived and supervised the study. Xinyu Ge and Kaijing Wang designed the experiments. Kaijing Wang, Yuanyuan Zhang, Xiaoxue Ma and Xinyu Ge performed the experiments. Kaijing Wang and Yuanyuan Zhang analysed the data. Kaijing Wang and Xinyu Ge wrote the manuscript. Yuanyuan Zhang, Xiaoxue Ma and Yewei Deng reviewed and edited the manuscript. All authors approved the final version of the manuscript and agree to be accountable for all aspects of the work in ensuring that questions related to the accuracy or integrity of any part of the work are appropriately investigated and resolved. All persons designated as authors qualify for authorship, and all those who qualify for authorship are listed.

## CONFLICT OF INTEREST

None declared.

## Supporting information

Statistical Summary Document

## Data Availability

The data that support the findings of this study are available from the corresponding author upon reasonable request.
